# The Effect of Notch and Molecular Weight on the Impact Fracture Behavior of Polycarbonate

**DOI:** 10.3390/polym16081072

**Published:** 2024-04-11

**Authors:** Xueting Xu, Tao Wang, Qiwei Sun, Bolun Wang, Yong Ge, Jianlin Lang, Yue Yan

**Affiliations:** 1 Beijing Institute of Aeronautical Materials, Beijing 100095, China; xuxueting00@163.com; 2 Beijing Engineering Research Center of Advanced Structural Transparence for the Modern Traffic System, Beijing 100095, China; stkidsqw@163.com (Q.S.); blwang10@foxmail.com (B.W.); sky_gy@126.com (Y.G.); langjl@126.com (J.L.); 3 Baimtec Material Co., Ltd., Beijing 100095, China

**Keywords:** molecular weight, V-shaped notch, polycarbonate, Charpy impact

## Abstract

The impact protection applications of polycarbonate (PC) products are gradually increasing. Due to the high sensitivity of PC to notches, research on notch impacts has become very important. In this paper, the impact performance of PC with two different molecular weights under different notch states was investigated. Three notch size factors, namely notch tip radius, notch angle, and notch center depth, were selected to design orthogonal experiments and research impact toughness. Subsequently, a single-factor study was conducted on the impact radius at the tip of the notch, which was the most important factor affecting the impact performance. Research shows that the brittle–ductile-transition tip radius of high-molecular-weight PC is 0.15 mm, and it has a higher impact toughness than low-molecular-weight PC during the brittle fracture process. The brittle–ductile-transition tip radius of lower molecular weight is 0.25 mm, while low-molecular-weight PC has a higher impact toughness during the ductile fracture process. The brittle and ductile fracture mechanisms of PC with different molecular weights were analyzed by observing the stress changes and cross-sectional morphology.

## 1. Introduction

PC is a glassy amorphous polymer, which has excellent transparency, high impact strength, and high heat resistance. PC is frequently used in impact protection applications [1], including aircraft canopies [2], face shields, goggles, and blast shields. Thus, the impact response of PC products [3,4] is a highly worthy research topic. Molecular weight is a key parameter that affects the physical, mechanical, and processable properties of polymers, and directly determines the application of the product, as well as PC. For example, low-molecular-weight PC is suitable as a substrate for digital storage media [5], and high-molecular-weight PC with excellent optical properties is used in aircraft windshields [6] and bulletproof shields [7]. 

As is well known, the mechanical behavior of PC exhibits notch sensitivity [8] and the actual usage environment of PC impact protection products is complex [9]. It is inevitable that small notches may occur due to collision and other reasons during the application process. Whether these notches will affect the impact performance of PC [10] is an important evaluation indicator. Polycarbonate is also used as a material for structural components that are usually complex in shape and subjected to severe mechanical loading. The presence of notches reduces the impact resistance of structural components because of the stress concentration. There are two forms of failure after impact: brittle fracture and ductile fracture [11]. PC is prone to transition from ductile fracture to brittle fracture in the presence of notches. The risk of brittle fracture is a very important evaluation factor in applications, as components may experience catastrophic failures at very low deformation levels. The research on the fracture behavior of PC after being impacted [12,13,14,15] is also a research focus.

The study of the molecular weight [16] and notch impact [17] of PC has received a great deal of attention from the scientific community in recent decades. Shotaro et al. [18] investigated the effects of molecular weight and heat treatment on the fracture of PC. The results showed that as the molecular weight increases, the toughness of PC increases because the higher molecular weight increases the stress of slippage for entangled molecular chains. Yoshitaka et al. [19] performed coarse-grained molecular dynamics (CGMD) simulations of PC and investigated how the state of entanglement influences the deformation behavior for simulation models with various molar masses. They found a significant effect of the molar mass on the stress–strain curves; i.e., the larger the molar mass is, the larger the stress that can be reached after yielding, suggesting that molar mass plays a major role in the brittle–ductile transition of PC. As for the notch fracture of PC, Aranda-Ruiz et al. [20] studied the dynamic three-point bending tests in a compression split Hopkinson pressure bar with different thicknesses and eccentricities of the initial notch of the test specimens. The results show that there is a transition in the failure mode of PC, which occurs under normal conditions of pressure and temperature. In addition, it has been shown that crack propagation starts earlier in the cases of brittle fracture than ductile fracture, and that crack propagation velocities are two orders of magnitude lower in the latter case. Anshul et al. [21] studied the brittle fracture of PC with different notch-tip radii under static and dynamic loading. They found that the mean stress required for defect nucleation increases with decreasing notch-tip radius due to increased triaxiality at the notch-tip. Defect initiation stresses are also higher for dynamic conditions compared to static loading. Defect initiation toughness for dynamic loading is always higher than that for static loading, but the reduction in defect initiation toughness with decreasing notch-tip is severe for dynamic loading. Kilwon et al. [22] researched the effect of notch radius on the impact behavior of PC and rubber-toughened PC, which is investigated by using a model based on the slip-lines field theory. Impact strength, determined by the Charpy impact test, was found to increase drastically with increasing notch radius for pure PC, whereas the increase in impact strength with increased notch radius was not as extreme for rubber-toughened PC. 

At present, most of the research on PC is focused on the impact properties of molecular weight or notches, and little attention is paid to the combined effect of molecular weight and notches. For this reason, two different-molecular-weight PCs are selected to research their impact fracture behavior under different notch factors and analyze the most important factor, notch tip radius. This paper summarizes the impact failure modes and fracture characteristics of PC notches and provides the direction of material selection for different applications of PC.

## 2. Materials and Methods

### 2.1. Materials

The raw materials used in the experiment were PC-A and PC-B. The melt mass flow rate (MFR), number-average molecular weight (*M*_n_), weight-average molecular weight (*M*_w_), and molecular weight distribution (*M*_w_/*M*_n_) are shown in Table 1.

### 2.2. Methods

#### 2.2.1. Sample Preparation

**Figure 1:**
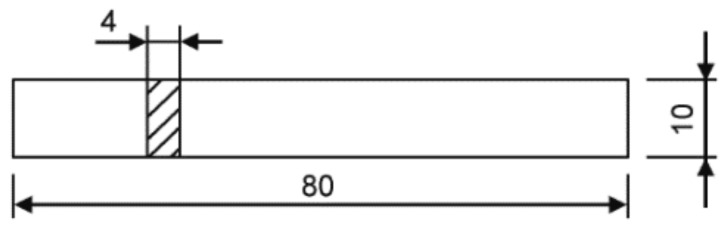
The figure of flexural and impact testing specimen.

The injection molding equipment adopted the CX130-750 injection molding machine of the Krauss Maffei (Hannover, Germany), and the screw diameter was 50 mm. The PC pellets were dried at 120 °C for 4 h, and the samples were prepared by injection molding. During the injection process, the melting temperature was 300 °C, the mold temperature was 100 °C, the injection speed was 10 mm·s^−1^, the holding pressure was 80 MPa, the holding time was 15 s, and the cooling time was 35 s. The size of the impact specimen is shown in Figure 1. 

#### 2.2.2. Orthogonal Experimental Design

Orthogonal experimental design is a quantitative design analysis method for studying multiple factors and levels. For multi-factor and multi-level experimental requirements, if experiments are conducted at each level of each factor, an excessive number of experiments will consume a lot of money and time. There are a few representative orthogonal experiments that can be selected where the factors that have the greatest impact on the results can be analyzed. Based on previous research [22,23] and the polycarbonate impact performance test standard (GB/T 1043.1-2008 [24]), we selected notch depth, notch tip radius, and notch angle to design orthogonal experiments. Each factor was subjected to orthogonal experiments at four levels, as shown in Table 2.

#### 2.2.3. Notch Processing

**Figure 2:**
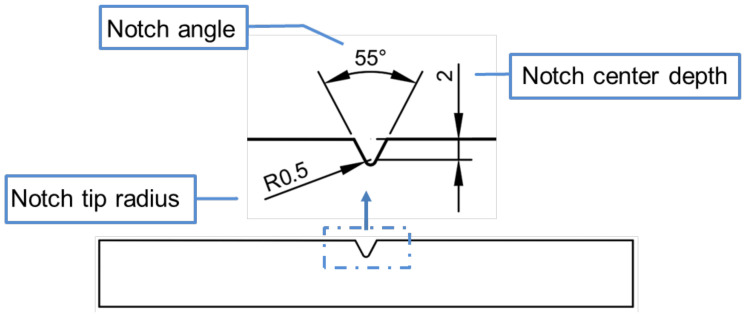
Charpy impact sample.

The sample was processed according to the parameters in the orthogonal experimental design table. The specimen with a 0.5 mm notch tip radius, 55° notch angle, and 2 mm notch center depth is shown in Figure 2

#### 2.2.4. Charpy Impact Test

A Charpy impact test was conducted using MTS company’s ZBC7501 (Eden Prairie, MN, USA) impact testing machine, and the test was conducted according to GB/T 1043.1-2008, with a pendulum energy of 15 J.

#### 2.2.5. Fracture Morphology Diagram

After the sample was subjected to a Charpy impact, the overall morphology of the cross-section was captured using a SONY Alpha 7C (Tokyo, Japan) camera, and the partial morphology of the cross-section was captured using an SU8020 scanning electron microscope (SEM) produced by Hitachi (Tokyo, Japan). The surface of the sample was sputtered with gold using HIYACHI company’s MC1000 (Tokyo, Japan).

#### 2.2.6. Residual Stress Testing

The residual stress at the sample notch was tested using the photoelastic method, and the test results were based on the optical path difference (σ). The testing equipment was the WPA-100-L wide-range 2D birefringence tester from LUCEO (Tokyo, Japan). The relationship between residual stress and optical path difference followed the stress optical law, expressed as

R = C d (σ_1_ − σ_2_), (1)

where R is the optical path difference, and C is the stress optical coefficient, which is an inherent property of the material. When the testing temperature is fixed, it can be considered that C is a fixed value; d is the thickness of the sample; and σ_1_ − σ_2_ is the difference in principal stress. Both C and d are constants, so the residual stress of the sample is linearly related to the optical path difference.

A real-time stress observation device was built using Instron’s 5982 universal testing machine, paired with a three-point bending fixture, with a testing rate of 20 mm/min to simulate the slow impact process. At the same time, stress polarization meters produced by Molding Innovation were placed on both sides of the sample to observe stress changes through the color changes of the sample stripes.

## 3. Results and Discussion

### 3.1. Orthogonal Experiment of Charpy Impact

The impact results of PC-A and PC-B on Charpy impact are shown in Table 3. To further analyze the influencing factors of notch impact, the range method was used to analyze the orthogonal experimental results, as shown in Table 4.

Range analysis can determine the primary and secondary order of the influence of each factor and determine the optimal process parameters. Therefore, this paper adopts the range analysis method to analyze the experimental data of impact toughness. Here, K_i_ is used to represent the sum of the corresponding experimental results when this factor is at the i level. k_i_ is the mean value of K_i_. 

The range R is R = max{k_1_, k_2_, k_3_, k_4_} − min{k_1_, k_2_, k_3_, k_4_}.

R represents the magnitude of variation in the experimental index when the level factor changes. When R is large, it means that this factor has a large effect on the evaluation index and is thus more important. Therefore, we can rely on R to rank the importance of the factors. 

**Figure 3:**
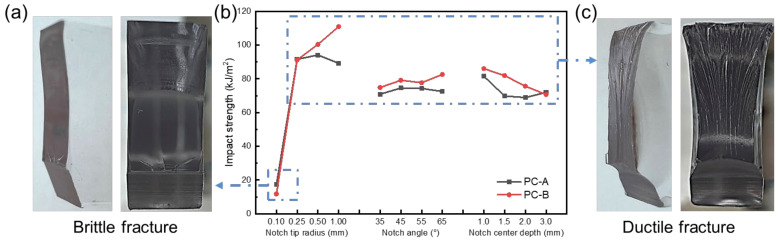
Impact toughness and cross-sectional diagram of orthogonal experiment: (**a**) cross-sectional diagram of brittle fracture; (**b**) effect of various factors on impact toughness; (**c**) cross-sectional diagram of ductile fracture.

According to the range analysis results, the influence degree of notch parameters on impact was as follows: notch tip radius > notch center depth > notch angle. According to the range analysis results, the chart of the change trend from the orthogonal test indexes with each experimental factor was drawn, as shown in Figure 3b. Obviously, the influence of notch tip radius on impact toughness was much greater than the influence of notch center depth and notch angle. NISITANI et al. [25] also had similar conclusions. As the notch radius increased, the impact toughness significantly increased. The angle of the notch had almost no effect on the impact toughness. It was worth noting that as the depth of the notch increased, the impact toughness only slightly decreased. The notch increased in depth, the length of the residual ligament shortened, and the energy required for damage also reduced, but its effect on the impact toughness was far less significant than the notch sharpness.

Based on the impact test results and cross-sectional morphology, it could be seen that the impact characteristics of PC were mainly divided into two types. Firstly, when the notch tip radius was 0.1 mm, the impact toughness was relatively low (about 15 kJ/m^2^). This was a brittle fracture, as shown in Figure 3a. The brittle fracture section was relatively smooth and flat, and there was an obvious crack source at the center of the notch tip and no necking on both sides of the section. Secondly, the radius of the notch tip was 0.25~1.00 mm, and the impact toughness was relatively high (about 70–120 kJ/m^2^), which was ductile fracture, as shown in Figure 3c. The morphology of the ductile fracture section was rough and torn. There was no obvious crack source in the center of the notch tip, and there was obvious necking on both sides of the section. When the specimen is impacted, the notch is in a tensile state. The response area at the tip of the sharp notch is small, and the PC cannot withstand the impact load, resulting in direct fracture and low impact toughness. The response area at the non-sharp notch tip is relatively large, and the sample fractures after yielding, producing plastic deformation to absorb impact energy. Therefore, it has higher impact toughness.

The pattern of impact behavior of PCs with different molecular weights as a function of notch geometry was the same. PC-B had a slightly higher impact toughness overall, while PC-A had a higher impact toughness in a few cases, e.g., 0.10 mm notch tip radius.

### 3.2. Single-Factor Test of Charpy Impact

#### 3.2.1. Charpy Impact Test Results and Cross-Sectional Morphology Analysis

**Figure 4:**
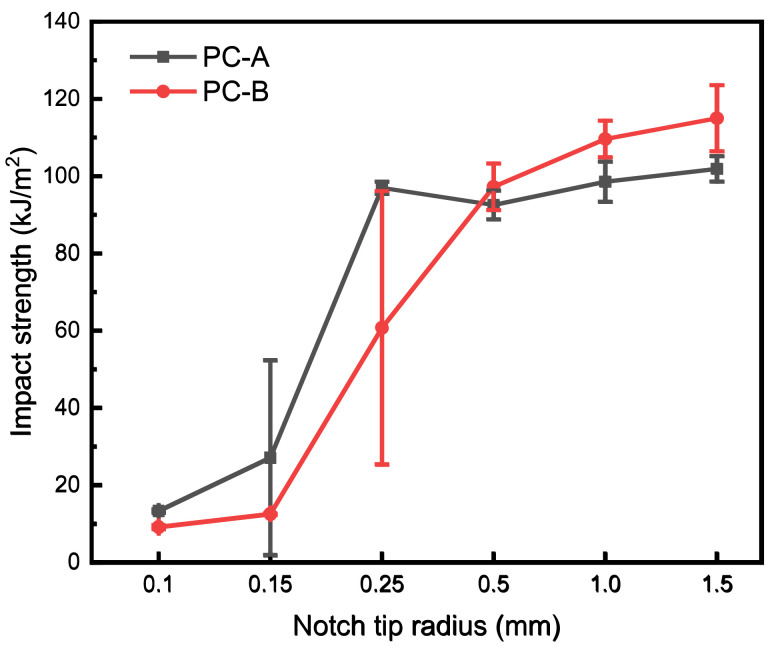
Impact toughness of single-factor test.

The radius of the notch tip is the most important factor affecting the impact toughness of PC notches and deserves further research. Single-factor studies were conducted on two types of PC with notch angles of 45°, a notch center depth of 2.0 mm, and notch tip radii of 0.10 mm, 0.15 mm, 0.25 mm, 0.50 mm, 1.00 mm, and 1.50 mm. The experimental results are shown in Figure 4. The impact toughness of both PC materials increased with the increase in the notch tip radius. Kilwon et al.’s [22] study also confirmed this pattern. C-A had higher impact toughness when the notch tip radius was less than 0.5 mm, while PC-B had higher impact toughness when the notch tip radius was 0.5 mm or more. 

**Figure 5:**
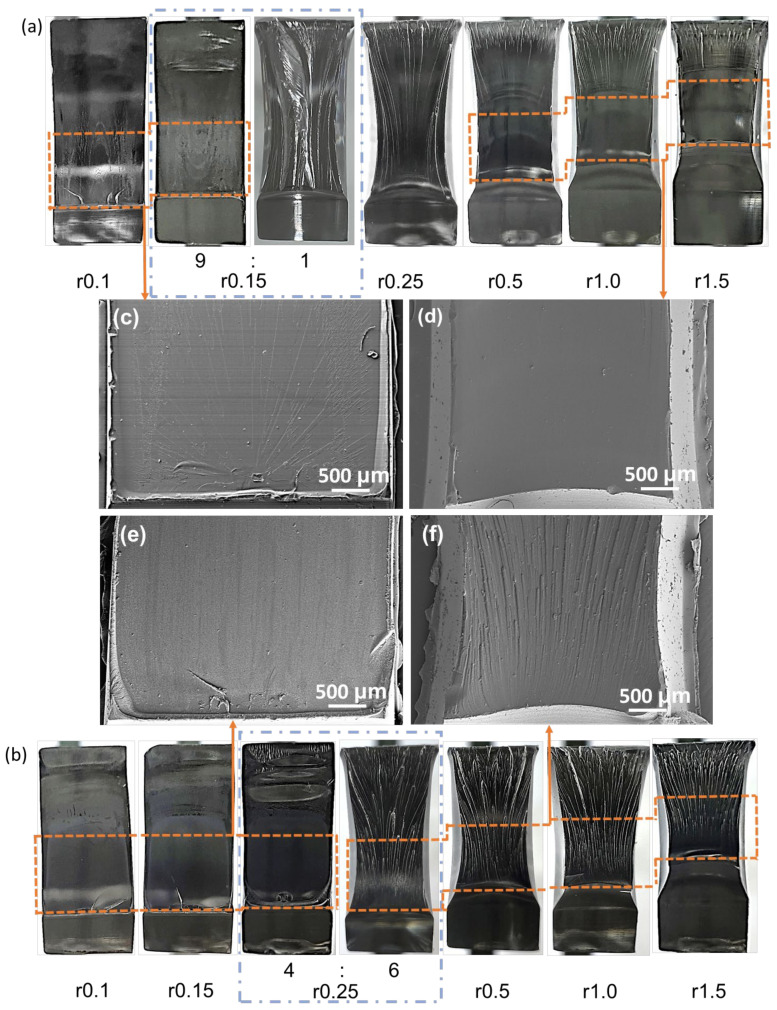
Cross-sectional diagram of single-factor Charpy impact: (**a**) PC-A; (**b**) PC-B; (**c**) crack initiation of PC-A brittle fracture; (**d**) crack initiation of PC-A ductile fracture; (**e**) crack initiation of PC-B brittle fracture; (**f**) crack initiation of PC-B ductile fracture.

The cross-section of the one-factor notched impact specimen is shown in Figure 5. Figure 5a shows the cross-section of PC-A with different notch tip radii, where the notch tip radius of 0.15 mm was the brittle toughness transition point. Figure 5b shows the cross-sectional view of PC-B with different notch tip radii, with a brittle–ductile transition point at a notch tip radius of 0.25 mm. The brittleness or toughness of the polymer fracture mode is closely related to its yield stress [26]. High-molecular-weight PC has a lower yield stress, which means it is easier to yield. The sample exhibits a yield state before fracture, and the impact toughness is higher. Therefore, PC-A transforms into ductile fracture in a sharper notch state.

The crack propagation characteristics of PC-A brittle fracture are shown in Figure 5c, and a slight ductile fracture mode can be observed. The crack propagation characteristic diagram of PC-B brittle fracture is shown in Figure 5e. By comparing the two molecular-weight cross-sections, it could be found that the fracture initiation of PC-B brittle fracture was smoother and completely presented the characteristic morphology of brittle fracture. PC-B had lower impact toughness in the sharp notched state due to its higher yield stress and lower entanglement degree. The crack propagation characteristics of PC-A ductile fracture are shown in Figure 5d. The crack propagation of PC-A specimens with a notch tip radius of 0.5 mm or more was relatively smooth and flat, with some brittle fracture morphology. The far end of the fracture showed a rough tearing pattern with low impact toughness. The crack propagation characteristics of PC-B ductile fracture are shown in Figure 5f, which is almost entirely the rough tearing mode. PC with lower molecular weight has a higher fracture strength and is more difficult to fracture after yielding, resulting in higher impact toughness in the non-sharp notch state. This is consistent with the results of impact toughness. The cross-sectional view confirms that PC-A has higher impact toughness under brittle fracture, while PC-B has higher impact toughness under ductile fracture.

#### 3.2.2. Stress Analysis

**Figure 6:**
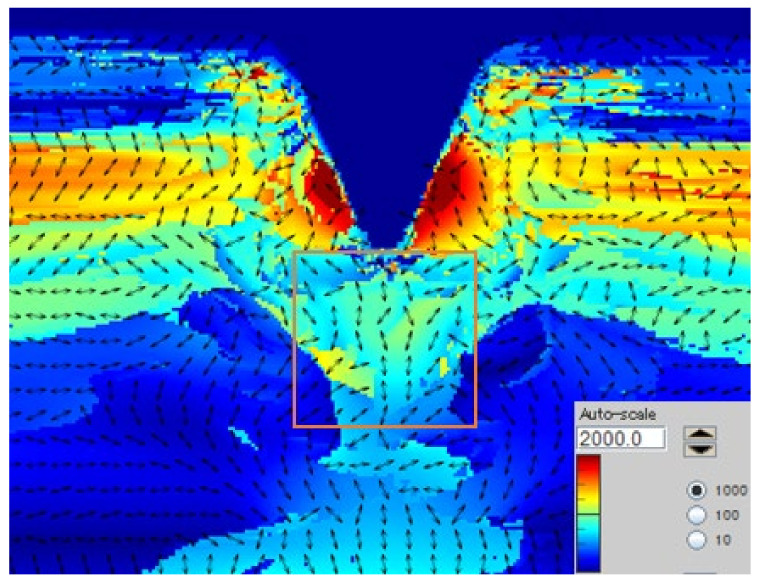
Residual stress measurement area.

**Figure 7:**
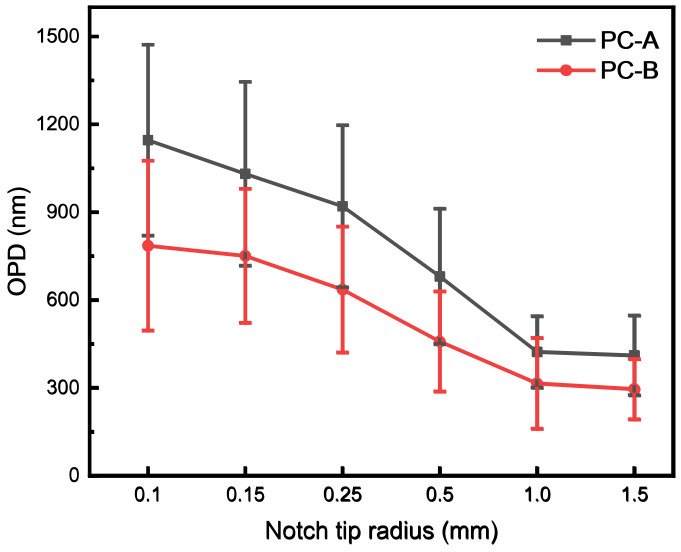
Residual stress of single-factor test.

**Figure 8:**
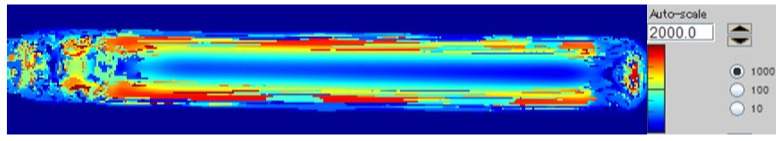
Residual stress of PC injection molded specimen.

The state of the notch tip is crucial to the impact performance. In order to analyze its influence mechanism, the residual stress at the notch tip was measured by the photoelastic method, and the measurement area is shown in Figure 6. The residual stress of PC is shown in Figure 7. As the radius of the notch tip increased, the residual stress of both PCs decreased. The stress distribution of the PC injection sample was higher at the edge and lower in the middle, as shown in Figure 8. The increase in radius means that the tip of the notch is closer to the middle, thereby reducing the residual stress. When the notch is sharp, the local stress is large, and the ability to withstand external forces is further reduced. After imposing external forces, it is easy to produce cracks, resulting in cracks and fractures. The residual stress of PC-A is always higher than that of PC-B. Due to their high entanglement density, the movement of high-molecular-weight PC fragments is difficult, and they are in a more unbalanced conformational state, resulting in higher residual stress. It is the tightly knit molecular chains that enable high-molecular-weight PC to better resist the impact in sharp notches. Low-molecular-weight PC has more stretchable molecular chains, a more uniform arrangement, and lower residual stress after injection molding. The uniform conformational arrangement is less likely to break under non-sharp impact plastic deformation, resulting in higher impact toughness.

**Figure 9:**
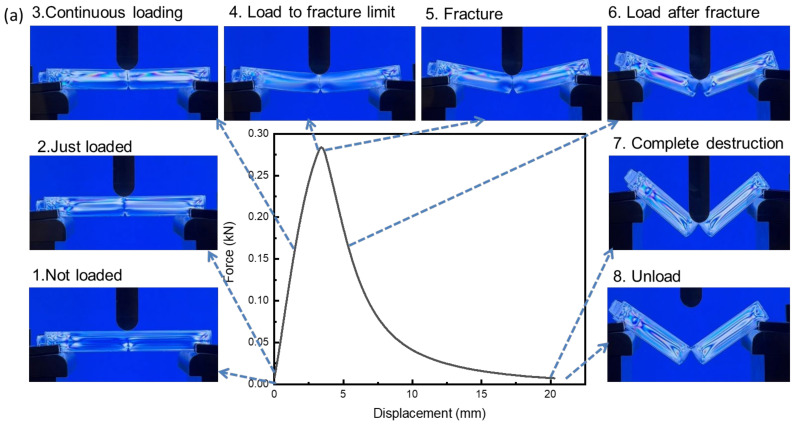

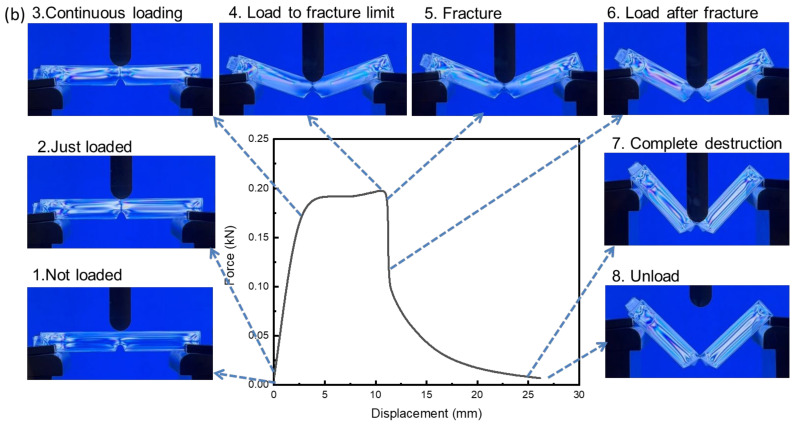
Stress change: (**a**) PC-A-r0.1; (**b**) PC-A-r1.5.

**Figure 10:**
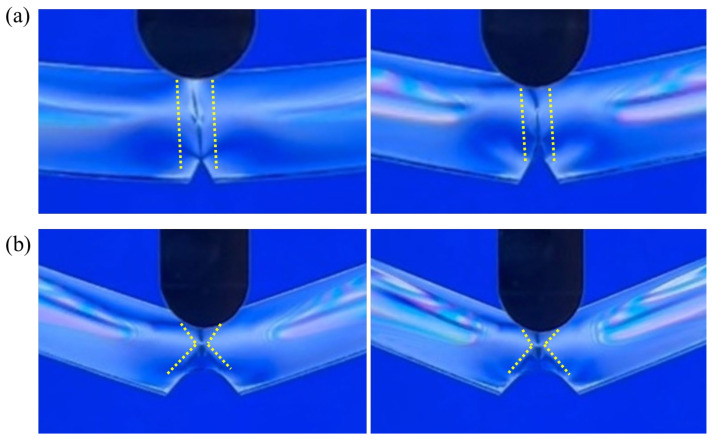
Stress characteristics of the notch zone before and after fracture: (**a**) PC-A-r0.1; (**b**) PC-A-r1.5.

The deformation of polymers under external forces is closely related to stress changes, and stress changes can be observed from the distribution of photoelastic stress stripes. The results are shown in Figure 9 with the stress variation of the PC-A specimen with a notch tip radius of 0.1 mm. When there was no initial force loading, the stress level of the specimen was relatively low and evenly distributed, with stress concentration at the notch. When the sample was subjected to the load, the stress increased, exhibiting an axisymmetric distribution. As the load continued to increase, the stress also increased, and the stress at the notch became more concentrated. The tip area was small and it was easy to form crazes. When the load increased to the point where the specimen could not withstand it, a fracture occurred from the notch, and the stress rapidly decreased at the moment of fracture. As the load increased, the crack propagated along the notch, and then the stress slowly decreased until the specimen could not withstand the load, meaning that the specimen had completely failed. After experiencing load action, the stress level increased compared to the initial state, and the stress distribution was high on both sides and low in the middle, similar to the initial state. Figure 9b shows the stress variation of the PC-A specimen with a tip radius of 1.5 mm, which is roughly the same as the variation trend of the tip radius of 0.1 mm. The largest difference was in the fracture zone, as can be clearly seen in Figure 10. Figure 10a shows a stress close-up of the notch area before and after the fracture of the PC-A sample with a notch tip radius of 0.1 mm, and Figure 10b shows a stress close-up of the notch area before and after the fracture of the PC-A sample with a notch tip radius of 1.5 mm. After applying a load to the specimen with a radius of 1.5 mm at the notch tip, a shear band generated by plastic deformation could be observed. As the load increased, the shear band gradually expanded until a fracture occurred at the notch. The fracture area was accompanied by the necking phenomenon. This is also the most direct difference between ductile fracture and brittle fracture, as shown by the dotted line in Figure 10. The above phenomenon is the same for PC-B. 

#### 3.2.3. Analysis of Notch Impact Fracture Mechanism

**Figure 11:**
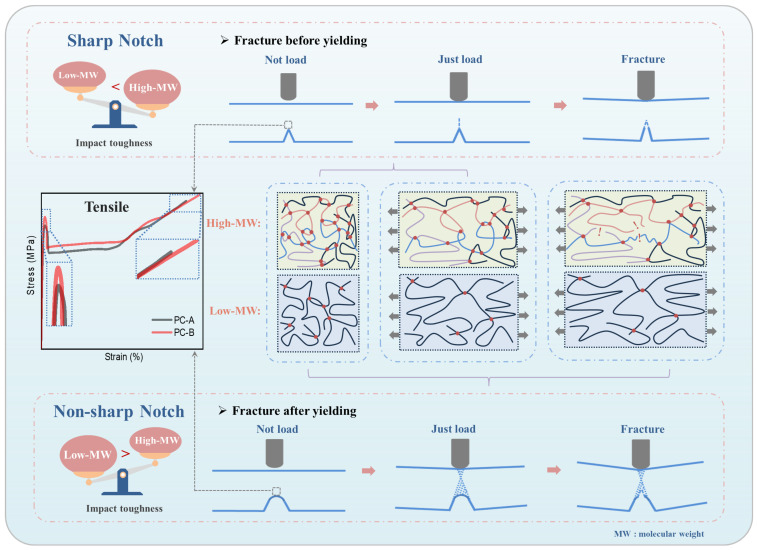
Notch fracture mechanism of high- and low-molecular-weight PC.

The impact fracture mechanism of a PC notch is shown in Figure 11. When the sample is impacted, the contact surface between the pendulum and the sample is in a compressed state, and the tip of the notch is in a stretched state [27]. For the sharp notch state, after the impact, the tip of the PC notch begins to crack, and then the crack is completely broken. This is a typical brittle fracture with a smooth cross-section. The sample fractures before yielding and the molecular chains at the tip notch are slightly stretched. High-molecular-weight PC has higher impact toughness due to its longer chain segments and tighter entanglement. From the stress–strain curve, it can be found that the yield stress of high-molecular-weight PC is lower, which is the reason why high-molecular-weight PC transforms into ductile fracture in a sharper notch state. In the non-sharp notch state, PC forms a shear band after being impacted, and then cracks appear in the notch, accompanied by plastic deformation throughout the process. This is a typical ductile fracture with a rough cross-section. The sample undergoes plastic deformation and fracture after yielding, and the molecular chains at the tip notch undergo more stretching. Due to excessive entanglement, high-molecular-weight PC is unable to freely stretch its molecular chains during the stretching process, resulting in more chain breaks [28], and the plastic deformation cannot respond in time. Meanwhile, the fracture strength and energy absorption of high-molecular-weight PC are lower, resulting in lower impact toughness.

## 4. Conclusions

In this work, the impact properties of two molecular-weight PCs with different notch sizes were tested, and the impact fracture mechanism was investigated. Firstly, a three-factor and four-level orthogonal impact test was designed, and it was found that the influence of notch tip radius on impact performance was much greater than those of the notch angle and the notch depth. The impact characteristics of PC are mainly divided into two types: one is brittle fracture with a smooth cross-section and low impact toughness, and the other is ductile fracture with a rough tearing pattern on the cross-section and high impact toughness. Subsequently, a single-factor impact test was carried out on the radius of the notch tip, and it was found that high-molecular-weight PC could transition from brittle fracture to ductile fracture under a sharper notch. Moreover, high-molecular-weight PC has a higher impact toughness in the brittle fracture process with small divergent patterns at the fracture initiation end, while lower-molecular-weight PC has a smooth cross-section. The lower molecular weight has higher impact toughness under non-sharp notches with rough tearing patterns on the cross-section, while higher molecular weight has a smoother fracture initiation and gradually transforms into a tearing pattern. By recording the stress state under low impact, the change in the shear band during the ductile fracture process of PC was observed, while brittle fractures did not show shear band formation.

## Figures and Tables

**Table 1:** MFR and molecular weight of PC.

PC	MFR (g/10 min)	*M*_n_ (g/mol)	*M*_w_ (g/mol)	*M*_w_/*M*_n_
PC-A	3.3	30,010	51,940	1.730
PC-B	10	23,400	38,190	1.632

**Table 2:** Orthogonal experimental design table.

	Factor	A Notch Tip Radius (mm)	B Notch Angle (°)	C Notch Center Depth (mm)
Level				
1		0.10	35	1.0
2		0.25	45	1.5
3		0.50	55	2.0
4		1.00	65	3.0

**Table 3:** Impact results.

Test Number	Notch Tip Radius (mm)	Notch Angle (°)	Notch Center Depth (mm)	PC-A Impact Toughness (kJ/m^2^)	PC-B Impact Toughness (kJ/m^2^)
1	0.10	35	1.0	13.8	12.1
2	0.10	45	1.5	13.8	11.6
3	0.10	55	2.0	10.5	11.4
4	0.10	65	3.0	31.1	12.0
5	0.25	35	1.5	93.6	91.8
6	0.25	45	1.0	105.7	105.9
7	0.25	55	3.0	80.7	79.7
8	0.25	65	2.0	86.4	87.5
9	0.50	35	2.0	91.0	96.8
10	0.50	45	3.0	90.9	92.1
11	0.50	55	1.0	114.4	104.2
12	0.50	65	1.5	80.2	108.6
13	1.00	35	3.0	85.1	98.8
14	1.00	45	2.0	87.8	107.1
15	1.00	55	1.5	91.7	115.7
16	1.00	65	1.0	92.3	122.3

**Table 4:** Range analysis of impact toughness.

Polycarbonate	Parameter	Notch Tip Radius (mm)	Notch Angle (°)	Notch Center Depth (mm)
PC-A	K_1_	69.3	283.4	326.2
	K_2_	366.4	298.3	279.3
	K_3_	376.5	297.4	275.7
	K_4_	356.9	290.0	287.9
	k_1_	17.3	70.9	81.6
	k_2_	91.6	74.6	69.8
	k_3_	94.1	74.3	68.9
	k_4_	89.2	72.5	72.0
	R	76.8	3.7	12.6
PC-B	K_1_	47.0	299.5	344.3
	K_2_	364.8	316.6	327.7
	K_3_	401.7	310.9	302.7
	K_4_	443.9	330.3	282.6
	k_1_	11.7	74.9	86.1
	k_2_	91.2	79.2	81.9
	k_3_	100.4	77.7	75.7
	k_4_	111.0	82.6	70.7
	R	99.2	7.7	15.4

## Data Availability

Data are contained within the article.
